# Two isostructural carbamates: the *o*-tolyl *N*-(pyridin-3-yl)carbamate and 2-bromo­phenyl *N*-(pyridin-3-yl)carbamate monohydrates

**DOI:** 10.1107/S2056989015019556

**Published:** 2015-10-24

**Authors:** Pavle Mocilac, John F. Gallagher

**Affiliations:** a School of Chemical Sciences, Dublin City University, Dublin 9, Ireland

**Keywords:** crystal structure, carbamate, hydrate, hydrogen bonding, isostructural, pyridine

## Abstract

The title carbamate monohydrates, C_13_H_12_N_2_O_2_·H_2_O and C_12_H_9_BrN_2_O_2_·H_2_O, form isomorphous crystals that are isostructural in their primary hydrogen-bonding modes. In both carbamates, the primary hydrogen bonding and aggregation involves cyclic amide–water–pyridine moieties as (N—H⋯O—H⋯N)_2_ dimers about inversion centres [as *R*_4_^4^(14) rings], where the participation of strong hydrogen-bonding donors and acceptors is maximized. The remaining water–carbonyl O—H⋯O=C inter­action extends the aggregation into two-dimensional planar sheets that stack parallel to the (100) plane. The Br derivative does not participate in halogen bonding. A weak intra­molecular C—H⋯O hydrogen bond is observed in each compound.

## Abstract 2

In the isostructural *ortho*-tolyl *N*-pyridinylcarbamate and *ortho*-bromo­phenyl *N*-pyridinylcarbamate monohydrates, the primary aggregation involves cyclic hydrogen bonding as (amide–water–pyridine)_2_ comprising _amide_N—H⋯O—H_water_⋯N_pyridine_ inter­actions about inversion centres [as 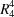
(14) rings]. The remaining H_2_O O—H donor and carbonyl O=C form a strong hydrogen bond. The participation of strong hydrogen-bonding donors and acceptors is maximized in short inter­actions, resulting in two-dimensional sheets.

## Chemical context   

Isomorphous crystals and isostructural compounds feature regularly in series of metalloorganic compounds, lanthanide derivatives as well as in halide-containing organics (*RX*, where *X* = F, Cl, Br, I and often including the methyl group, Me). Given the vast array of data available in the Cambridge Structural Database (CSD; Groom & Allen, 2014 ▸), the relative proportion of isostructural relationships between sets of crystal structures can readily be ascertained. As such, Oswald & Crichton (2009 ▸) have reported on the regularity with which chlorine (Cl) and methyl (Me) groups exhibit isostructurality based on analysis of pairs of compounds in the CSD (van de Streek & Motherwell, 2005 ▸), whereby an estimate of 25–30% of compound pairs are isostructural. In addition, Polito *et al.* (2008 ▸) have rationalized the differences and similarities between *ortho*-chloro and *ortho*-methyl­benzoic acids, while the ability of bromines (as Br—C) as well as other halogens to form isostructural pairs/series with methyl groups is well documented (Capacci-Daniel *et al.*, 2008 ▸).

These researchers have reported an elegant example of an isostructural series of 1,3-bis­(*meta*-dihalophen­yl)ureas (with halo = Cl, Br, I) that form isomorphous crystals in space group *P*2_1_2_1_2, (No. 18) and reported with mono- and di-tolyl analogues (Capacci-Daniel *et al.*, 2008 ▸). The mol­ecules associate *via* (N-H)_2_⋯O=C inter­actions into 1D chains [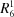
(6) motif] and with π–π stacking inter­actions and halogen contacts completing the aggregation. One can surmise that isostructural series in organic mol­ecules are possible whereby 1-2 strong hydrogen bonds dominate the inter­actions and drive mol­ecular association, despite often semi-effective cumulative competition from other inter­actions, whilst taking into account the effect of atom/group replacement (Groom & Allen, 2014 ▸).

Further examples in coordination chemistry include the halogen-substituted pseudoterpyridine Zn^II^ homoleptic mononuclear complexes that lack strong hydrogen bonding and with the packing relying on a subtle inter­play of weaker inter­actions, where isostructurality is rare amongst the four (F/Cl/Br/I) halogens (Dumitru *et al.*, 2013 ▸). Another example is where the metal complexes (Co^II^, Ni^II^, Cu^II^, Zn^II^) form an isostructural series when coordinated to a tetra­aryl­aza­dipyromethene ligand (Palma *et al.*, 2009 ▸). The inter­changeability effects of C—H and C—F groups in series of isomeric fluorinated benzamides has been noted (Chopra & Guru Row, 2008 ▸; Donnelly *et al.*, 2008 ▸) and for C—H/C—CH_3_ (Mocilac *et al.*, 2010 ▸). More recently, Gomes and co-workers have reported four *N*-(4-halophen­yl)-4-oxo-4*H*-chromene-3-carboxamides (halo = F/Cl/Br/I), where isostructural (F/Cl) and (Br/I) pairs are noted though all four compounds have similar supra­molecular structures (Gomes *et al.*, 2015 ▸).
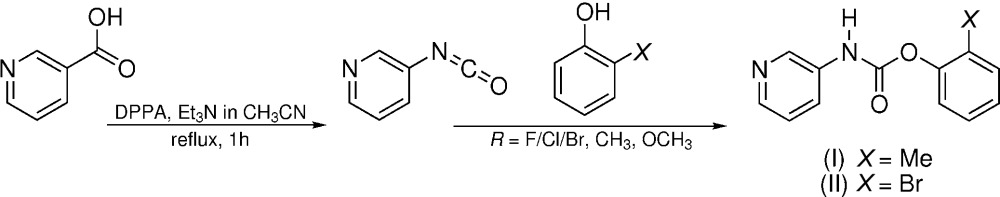


## Structural commentary   

The carbamates synthesised from condensation reactions (shown in the scheme) as their methyl (**CmoM**) and bromo-derivatives (**CmoBr**) crystallize as isostructural monohydrates. The differences between the unit-cell parameters (*a*, *b*, *c, β*) are < 1% for **CmoM** (I) and **CmoBr** (II). Both mol­ecules have similar geometric data (bond lengths and angles) apart from the (*ortho*)C—CH_3_/Br bond-length differences and some inter­planar data. The mol­ecules have three primary torsion angles along the mol­ecular backbone namely _benzene_C—C—O—C, C—O—C—N and C—N—C—C_pyridine_ where the mol­ecule can adopt one of several conformations in solution. In (I) and (II), both aromatic rings are twisted from co-planarity with the four-membered OCON non-H carbamate atom backbone. The **CmoM** C_6_ ring is oriented at an angle of 87.83 (4)° to the central carbamate moiety which lies at an angle of 25.79 (7)° to the C_5_N ring; the corresponding data for **CmoBr** are 88.60 (11) and 26.67 (18)° and highlighting the similarities in the two mol­ecular structures. For comparison, we have previously reported an isomer grid of nine related meth­oxy­carbamates (**C*xx*OMe**) (***x*** = *ortho*-/*meta*-*/para*-) in order to compare their crystal structures and mol­ecular models (Mocilac & Gallagher, 2013 ▸).

**Figure 1:**
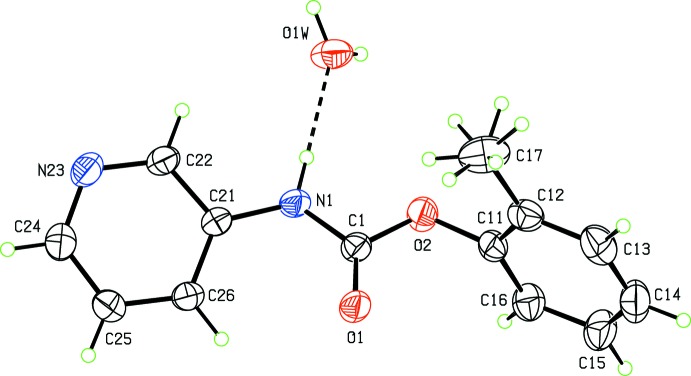
View of the asymmetric unit of (I)·H_2_O, showing the atomic numbering schemes. Rotational disorder of the methyl group is depicted. Displacement ellipsoids are drawn at the 30% probability level.

**Figure 2:**
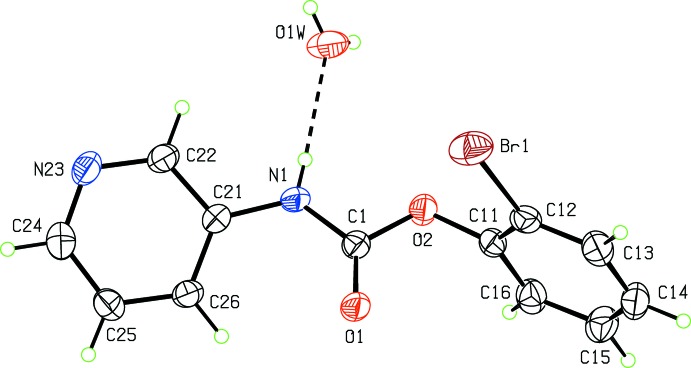
View of the asymmetric unit of (II)·H_2_O, showing the atomic numbering schemes. Displacement ellipsoids are drawn at the 30% probability level.

In the **C*xx*OMe** series (Mocilac & Gallagher, 2013 ▸), the primary inter­action mode for all nine isomers is the amide⋯pyridine (as N—H⋯N) and typically aggregating as catemers, dimers or trimers. However, there is no evidence for the familiar N—H⋯O=C (amide⋯amide) type hydrogen bonding (Mocilac & Gallagher, 2013 ▸). This is in comparison to a series of related benzamides/carboxamides containing one strong donor/two strong acceptors where competition arises resulting in the formation of either (i) N—H⋯N or (ii) N—H⋯O=C hydrogen bonds as the primary strong inter­action (Mocilac *et al.*, 2010 ▸, 2012 ▸). In the title structures of **CmoM** (Fig. 1 ▸) and **CmoBr** (Fig. 2 ▸), the presence of a water mol­ecule in the asymmetric unit was unexpected (water typically assists in the decomposition of organic carbamates at room temperature) though it can be shown to confer additional stability on the structure by forming compact hydrogen bonding and contributing to sheet formation. The retention of carbamate crystal structure integrity is observed over time (as measured in months).

## Supra­molecular features   

**Figure 3:**
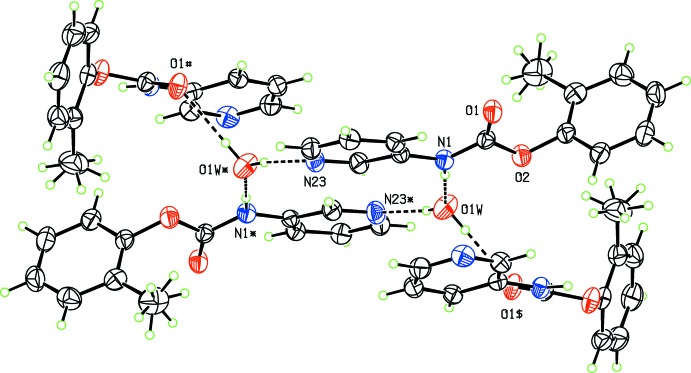
Part of the crystal structure of (I) with the primary inter­actions as a hydrogen-bonded moiety of four carbamates surrounding two hydrogen-bonded water mol­ecules and with selected labels. The symmetry-related mol­ecules with suffices *, #, $ are positioned at (1 − *x*, 2 − *y*, −*z*), (1 − *x*, 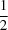 + *y*, −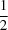 − *z*) and (*x*, 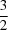 − *y*, 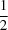 + *z*), respectively.

**Figure 4:**
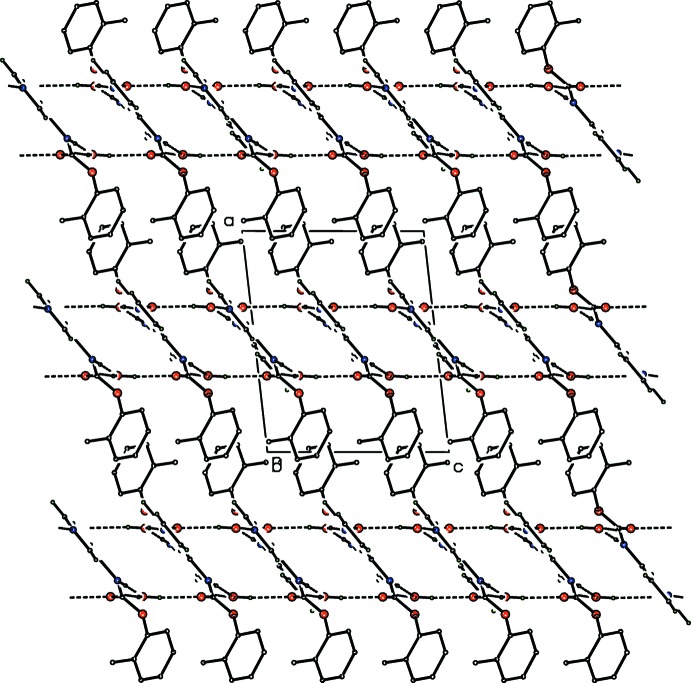
A packing diagram of the two-dimensional sheets and inter­locking *o*-tolyl groups in **CmoM** (with aromatic C_6_ H atoms removed for clarity). Atoms are drawn as spheres of an arbitrary size.

The three primary hydrogen bonds in (I) and (II) as N1—H1⋯O1*W*, O1*W*—H1*W*⋯O1 and O1*W—*H2*W*⋯N23 (Tables 1 ▸ and 2 ▸) are classed as strong classical hydrogen bonds with donor–acceptor (*D*⋯*A*) distances < 2.95 Å and *D*—H⋯*A* angles close to linearity at 180°. In Figs. 3 ▸–5 ▸ ▸ the crystal packing and inter­actions for **CmoM** are shown and in general are similar for **CmoBr**. The amide⋯water⋯pyridine hydrogen bonds facilitate aggregation of a centrosymmetric ring of hydrogen bonds [as 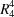
(14) rings] (Fig. 3 ▸) which, when combined with the water⋯amide carbonyl (O=C) inter­action, generates a compact flattened 2D sheet of hydrogen bonds that lies parallel to the (100) plane (Figs. 4 ▸ and 5 ▸). The hydrogen bonding inter­cepts the *a*-axis at 0.33 and 0.67 along the unit-cell axis and the sheet is a unit-cell length (*a*) in thickness with hydro­phobic aromatic rings at the 2D sheet surfaces. The 3D crystal structure arises where 2D sheets stack parallel to the *a*-axis direction.

## Synthesis and crystallisation   

Carbamate formation (**Cmo** ***X***; ***X*** = Me, Br): The simplest method of phenyl-*N*-pyridinyl-carbamate (**C*xx*** ***R***) synthesis is a condensation reaction of amino­pyridines with commercially available phenyl­chloro­formates in the presence of base (Et_3_N) and solvent (CH_2_Cl_2_). This is performed in an analogous fashion to the Schotten–Baumann reaction and can provide relatively pure products in high yields. However, when using 2-amino­pyridines, additional double carbamates are formed where both of the N—H H atoms are replaced by formates. In order to minimize double carbamate formation for these derivatives, reactions are usually performed by mixing the reagents without solvent and base at lower temperature, followed by simple recrystallization.

Another viable route into carbamate chemistry is to use an agent that transforms phenols into the required chloro­formate; however, a simpler and more straightforward method for carbamate synthesis is the Curtius rearrangement reaction (or Curtius reaction or degradation) involving the rearrangement of an acyl azide to an iso­cyanate. The acyl azide (in this case pyridinyl azide) can be formed from the carb­oxy­lic acid by a suitable agent like di­phenyl­phosphoryl azide. The acid can be easily converted to pyridinyl azides using di­phenyl­phosphoryl azide and at higher temperature (343 K) in the presence of base. The pyridinyl azides rearrange into pyridinyl iso­cyanates and following reaction with a phenol, the required phenyl-*N*-pyridinyl-carbamate (**C*xx*** ***R***) is generated.

**Reaction procedure:** A mixture of isonicotinic acid (1.2877 g, 10.46 mmol), Et_3_N (1.46 ml, 10.46 mmol), and di­phenyl­phosphoryl azide (2.258 ml, 10.46 mmol) was stirred for 1 h in 30 mL of dry aceto­nitrile at room temperature. The reaction mixture was carefully heated (water bath) to reflux for 1 h, then with 2-methyl­phenol or 2-bromo­phenol (10.46 mmol) added and the resulting solution heated at reflux temperatures for 7 h, gradually cooled and stirred overnight. If a white precipitate formed, it was filtered, washed with aceto­nitrile and dried (and usually found to be the pure product). The solvent was removed from the reaction mixture under reduced pressure, the residue dissolved in CH_2_Cl_2_, washed thrice with a solution of KHCO_3_ and Na_2_CO_3_ (pH = 9) and twice with brine/ammonium chloride (pH = 5). The organic fraction was removed *in vacuo* and the compound recrystallized from diethyl ether and CH_2_Cl_2_. If necessary, purification was accomplished by column chromatography using silica as the stationary phase and a mixture of CH_2_Cl_2_ and methanol (8:1) as mobile phase. Both **ComM** (46% yield, m.p. range = 352–357 K) and **ComBr** (21% yield, m.p. range = 359.2–359.9 K) compounds were obtained using this method (Mocilac, 2012 ▸).

## Refinement   

Crystal data, data collection and structure refinement details are summarized in Table 3 ▸. The refinement of structures (I) and (II) were performed similarly. H atoms attached to C atoms were treated as riding using the *SHELXL2014* (Sheldrick, 2015 ▸) defaults at 294 (1) K with C—H = 0.93 Å (aromatic) and *U*_iso_(H) = 1.2*U*_eq_(C) (aromatic). The methyl C—H = 0.96 Å (aliphatic) and *U*_iso_(H) = 1.5*U*_eq_(C). The amino N—H and water O—H H atoms were refined with isotropic displacement parameters in both structures (I) and (II). In (I) the methyl group H atoms were refined as disordered over two sets of sites with equal occupancies 60° apart.

## Supplementary Material

Crystal structure: contains datablock(s) global, CmoM, CmoBr. DOI: 10.1107/S2056989015019556/lh5787sup1.cif

Structure factors: contains datablock(s) CmoM. DOI: 10.1107/S2056989015019556/lh5787CmoMsup2.hkl

Structure factors: contains datablock(s) CmoBr. DOI: 10.1107/S2056989015019556/lh5787CmoBrsup3.hkl

Supporting information file. DOI: 10.1107/S2056989015019556/lh5787CmoMsup4.cml

Supporting information file. DOI: 10.1107/S2056989015019556/lh5787CmoBrsup5.cml

CCDC references: 1431472, 1431471

Additional supporting information:  crystallographic information; 3D view; checkCIF report

## Figures and Tables

**Figure 5:**
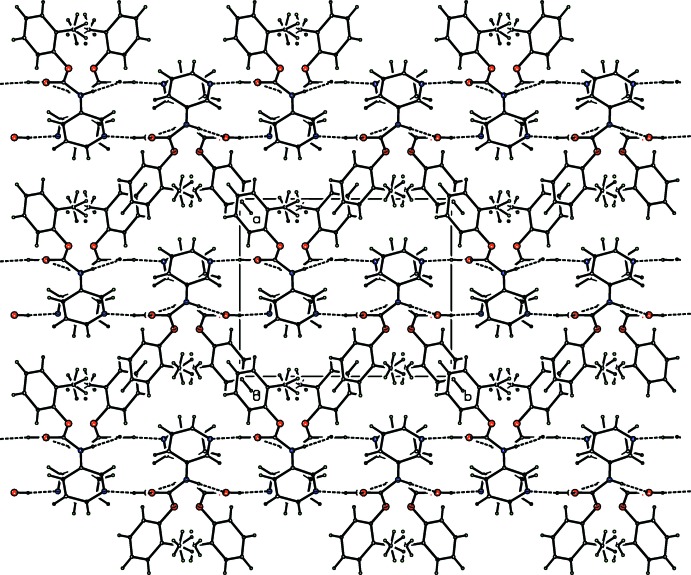
A packing diagram of **CmoM** as two-dimensional sheets as viewed orthogonal to the direction shown in Fig. 4 ▸. Atoms are drawn as spheres of an arbitrary size with all H atoms included.

**Table 1:** Hydrogen-bond geometry (Å, °) for **CmoM** [Chem scheme1]

*D*—H⋯*A*	*D*—H	H⋯*A*	*D*⋯*A*	*D*—H⋯*A*
N1—H1⋯O1*W*	0.875 (16)	1.953 (16)	2.8274 (14)	179.1 (15)
O1*W*—H1*W*⋯O1^i^	0.85 (2)	2.06 (2)	2.9126 (15)	173.2 (18)
O1*W*—H2*W*⋯N23^ii^	0.86 (2)	1.97 (3)	2.8266 (16)	170.6 (19)
C26—H26⋯O1	0.93	2.43	2.9337 (15)	114

Symmetry codes: (i) 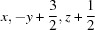; (ii) 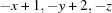.

**Table 2:** Hydrogen-bond geometry (Å, °) for **CmoBr** [Chem scheme1]

*D*—H⋯*A*	*D*—H	H⋯*A*	*D*⋯*A*	*D*—H⋯*A*
N1—H1⋯O1*W*	0.74 (3)	2.10 (3)	2.832 (4)	177 (3)
O1*W*—H1*W*⋯O1^i^	0.75 (4)	2.18 (5)	2.924 (4)	177 (5)
O1*W*—H2*W*⋯N23^ii^	0.71 (4)	2.13 (4)	2.837 (4)	175 (4)
C26—H26⋯O1	0.93	2.44	2.946 (4)	114

Symmetry codes: (i) 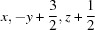; (ii) 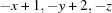.

**Table 3:** Experimental details

	**CmoM**	**CmoBr**
Crystal data		
Chemical formula	C_13_H_12_N_2_O_2_·H_2_O	C_12_H_9_BrN_2_O_2_·H_2_O
*M*_r_	246.26	311.14
Crystal system, space group	Monoclinic, *P*2_1_/*c*	Monoclinic, *P*2_1_/*c*
Temperature (K)	294	294
*a*, *b*, *c* (Å)	10.9754 (2), 12.9877 (2), 8.9544 (2)	10.9036 (4), 13.0518 (3), 8.9804 (3)
β (°)	96.546 (2)	96.460 (3)
*V* (Å^3^)	1268.09 (4)	1269.90 (7)
*Z*	4	4
Radiation type	Mo *K*α	Mo *K*α
μ (mm^−1^)	0.09	3.24
Crystal size (mm)	0.61 × 0.36 × 0.19	0.35 × 0.20 × 0.04

Data collection		
Diffractometer	Agilent Xcalibur Sapphire3 Gemini Ultra	Agilent Xcalibur Sapphire3 Gemini Ultra
Absorption correction	Analytical (*ABSFAC*; Clark & Reid, 1998 ▸)	Analytical (*ABSFAC*; Clark and Reid, 1998 ▸)
*T*_min_, *T*_max_	0.962, 0.983	0.398, 0.844
No. of measured, independent and observed [*I* > 2σ(*I*)] reflections	14095, 4060, 2987	9904, 2811, 1881
*R*_int_	0.017	0.029
(sin θ/λ)_max_ (Å^−1^)	0.739	0.658

Refinement		
*R*[*F* ^2^ > 2σ(*F* ^2^)], *wR*(*F* ^2^), *S*	0.049, 0.136, 1.03	0.046, 0.106, 1.03
No. of reflections	4060	2811
No. of parameters	177	175
H-atom treatment	H atoms treated by a mixture of independent and constrained refinement	H atoms treated by a mixture of independent and constrained refinement
Δρ_max_, Δρ_min_ (e Å^−3^)	0.21, −0.16	0.68, −0.64

Computer programs: *CrysAlis PRO* (Agilent, 2012 ▸), *SHELXS97* (Sheldrick, 2008 ▸), *SHELXL2014* (Sheldrick, 2015 ▸) and *PLATON* (Spek, 2009 ▸).

## supplementary crystallographic information

### (CmoM) 2-Methylphenyl N-(pyridin-3-yl)carbamate monohydrate Crystal data

**Table:**

C_13_H_12_N_2_O_2_·H_2_O	*D*_x_ = 1.290 Mg m^−^^3^
*M**_r_* = 246.26	Melting point: 355 K
Monoclinic, *P*2_1_/*c*	Mo *K*α radiation, λ = 0.71073 Å
*a* = 10.9754 (2) Å	Cell parameters from 6097 reflections
*b* = 12.9877 (2) Å	θ = 2.4–31.5°
*c* = 8.9544 (2) Å	µ = 0.09 mm^−^^1^
β = 96.546 (2)°	*T* = 294 K
*V* = 1268.09 (4) Å^3^	Block, colourless
*Z* = 4	0.61 × 0.36 × 0.19 mm
*F*(000) = 520	

### (CmoM) 2-Methylphenyl N-(pyridin-3-yl)carbamate monohydrate Data collection

**Table:**

Agilent Xcalibur Sapphire3 Gemini Ultra diffractometer	*R*_int_ = 0.017
Radiation source: Enhance (Mo) X-ray Source	θ_max_ = 31.7°, θ_min_ = 2.4°
ω scans	*h* = −15→15
Absorption correction: analytical (ABSFAC; Clark & Reid, 1998)	*k* = −18→19
*T*_min_ = 0.962, *T*_max_ = 0.983	*l* = −12→12
14095 measured reflections	6097 standard reflections every 60 min
4060 independent reflections	intensity decay: 1%
2987 reflections with *I* > 2σ(*I*)	

### (CmoM) 2-Methylphenyl N-(pyridin-3-yl)carbamate monohydrate Refinement

**Table:**

Refinement on *F*^2^	Secondary atom site location: inferred from neighbouring sites
Least-squares matrix: full	Hydrogen site location: mixed
*R*[*F*^2^ > 2σ(*F*^2^)] = 0.049	H atoms treated by a mixture of independent and constrained refinement
*wR*(*F*^2^) = 0.136	*w* = 1/[σ^2^(*F*_o_^2^) + (0.0573*P*)^2^ + 0.1872*P*] where *P* = (*F*_o_^2^ + 2*F*_c_^2^)/3
*S* = 1.03	(Δ/σ)_max_ < 0.001
4060 reflections	Δρ_max_ = 0.21 e Å^−^^3^
177 parameters	Δρ_min_ = −0.16 e Å^−^^3^
0 restraints	Extinction correction: SHELXL2014 (Sheldrick, 2015), Fc^*^=kFc[1+0.001xFc^2^λ^3^/sin(2θ)]^-1/4^
Primary atom site location: structure-invariant direct methods	Extinction coefficient: 0.017 (2)

### (CmoM) 2-Methylphenyl N-(pyridin-3-yl)carbamate monohydrate Special details

**Table:**

Geometry. All e.s.d.'s (except the e.s.d. in the dihedral angle between two l.s. planes) are estimated using the full covariance matrix. The cell e.s.d.'s are taken into account individually in the estimation of e.s.d.'s in distances, angles and torsion angles; correlations between e.s.d.'s in cell parameters are only used when they are defined by crystal symmetry. An approximate (isotropic) treatment of cell e.s.d.'s is used for estimating e.s.d.'s involving l.s. planes.

### (CmoM) 2-Methylphenyl N-(pyridin-3-yl)carbamate monohydrate Fractional atomic coordinates and isotropic or equivalent isotropic displacement parameters (Å^2^)

**Table:**

	*x*	*y*	*z*	*U*_iso_*/*U*_eq_	Occ. (<1)
O1	0.34460 (9)	0.58485 (7)	0.04485 (11)	0.0602 (3)	
O2	0.26595 (9)	0.69019 (7)	0.21023 (10)	0.0605 (3)	
N1	0.41760 (10)	0.74813 (7)	0.09353 (12)	0.0489 (2)	
H1	0.3953 (14)	0.8055 (13)	0.1339 (17)	0.064 (4)*	
C1	0.34402 (11)	0.66676 (9)	0.10863 (12)	0.0468 (3)	
C11	0.17108 (12)	0.61974 (9)	0.22454 (13)	0.0507 (3)	
C12	0.06160 (14)	0.63221 (11)	0.13440 (16)	0.0606 (3)	
C13	−0.03242 (14)	0.56479 (14)	0.1608 (2)	0.0744 (4)	
H13	−0.1081	0.5705	0.1030	0.089*	
C14	−0.01636 (16)	0.49030 (14)	0.2696 (2)	0.0774 (5)	
H14	−0.0806	0.4461	0.2844	0.093*	
C15	0.09395 (16)	0.48072 (14)	0.35639 (18)	0.0750 (4)	
H15	0.1048	0.4302	0.4303	0.090*	
C16	0.18854 (14)	0.54588 (12)	0.33415 (15)	0.0623 (3)	
H16	0.2638	0.5400	0.3929	0.075*	
C17	0.0470 (2)	0.71323 (15)	0.0145 (2)	0.0997 (6)	
H17A	−0.0379	0.7320	−0.0053	0.150*	0.5
H17B	0.0751	0.6868	−0.0757	0.150*	0.5
H17C	0.0944	0.7727	0.0477	0.150*	0.5
H17D	0.1256	0.7290	−0.0168	0.150*	0.5
H17E	0.0126	0.7742	0.0535	0.150*	0.5
H17F	−0.0067	0.6883	−0.0699	0.150*	0.5
C21	0.51507 (11)	0.75035 (8)	0.00573 (12)	0.0429 (2)	
C22	0.55366 (12)	0.84604 (9)	−0.03989 (15)	0.0537 (3)	
H22	0.5115	0.9040	−0.0127	0.064*	
N23	0.64700 (11)	0.85974 (9)	−0.11985 (14)	0.0617 (3)	
C24	0.70646 (13)	0.77623 (12)	−0.15846 (16)	0.0607 (3)	
H24	0.7719	0.7844	−0.2148	0.073*	
C25	0.67509 (12)	0.67857 (11)	−0.11843 (15)	0.0563 (3)	
H25	0.7187	0.6221	−0.1477	0.068*	
C26	0.57875 (11)	0.66476 (9)	−0.03472 (14)	0.0494 (3)	
H26	0.5569	0.5991	−0.0059	0.059*	
O1W	0.34506 (12)	0.93442 (8)	0.22084 (13)	0.0712 (3)	
H1W	0.3406 (17)	0.9329 (15)	0.315 (3)	0.090 (6)*	
H2W	0.3436 (19)	0.9991 (19)	0.199 (2)	0.098 (6)*	

### (CmoM) 2-Methylphenyl N-(pyridin-3-yl)carbamate monohydrate Atomic displacement parameters (Å^2^)

**Table:**

	*U*^11^	*U*^22^	*U*^33^	*U*^12^	*U*^13^	*U*^23^
O1	0.0760 (6)	0.0421 (4)	0.0675 (6)	−0.0105 (4)	0.0304 (5)	−0.0123 (4)
O2	0.0724 (6)	0.0521 (5)	0.0617 (5)	−0.0120 (4)	0.0284 (4)	−0.0170 (4)
N1	0.0595 (6)	0.0356 (5)	0.0533 (5)	−0.0022 (4)	0.0133 (4)	−0.0054 (4)
C1	0.0567 (7)	0.0414 (5)	0.0437 (5)	−0.0015 (5)	0.0117 (5)	−0.0025 (4)
C11	0.0572 (7)	0.0487 (6)	0.0496 (6)	−0.0016 (5)	0.0207 (5)	−0.0101 (5)
C12	0.0676 (8)	0.0550 (7)	0.0601 (7)	0.0080 (6)	0.0111 (6)	−0.0065 (6)
C13	0.0552 (8)	0.0863 (11)	0.0819 (10)	0.0024 (7)	0.0083 (7)	−0.0138 (9)
C14	0.0702 (10)	0.0854 (11)	0.0825 (11)	−0.0174 (8)	0.0339 (9)	−0.0078 (9)
C15	0.0843 (11)	0.0795 (10)	0.0656 (9)	−0.0082 (8)	0.0276 (8)	0.0120 (8)
C16	0.0634 (8)	0.0722 (9)	0.0528 (7)	−0.0014 (7)	0.0129 (6)	0.0032 (6)
C17	0.1206 (16)	0.0748 (11)	0.0988 (13)	0.0181 (11)	−0.0091 (12)	0.0172 (10)
C21	0.0482 (6)	0.0383 (5)	0.0414 (5)	−0.0027 (4)	0.0020 (4)	0.0006 (4)
C22	0.0605 (7)	0.0393 (6)	0.0615 (7)	−0.0022 (5)	0.0072 (6)	0.0037 (5)
N23	0.0640 (7)	0.0536 (6)	0.0683 (7)	−0.0091 (5)	0.0106 (5)	0.0122 (5)
C24	0.0538 (7)	0.0690 (8)	0.0600 (7)	−0.0046 (6)	0.0103 (6)	0.0062 (6)
C25	0.0521 (7)	0.0564 (7)	0.0606 (7)	0.0061 (5)	0.0074 (5)	−0.0022 (6)
C26	0.0531 (6)	0.0403 (5)	0.0544 (6)	0.0012 (5)	0.0047 (5)	0.0023 (5)
O1W	0.1129 (9)	0.0451 (5)	0.0581 (6)	0.0132 (5)	0.0206 (6)	0.0011 (4)

### (CmoM) 2-Methylphenyl N-(pyridin-3-yl)carbamate monohydrate Geometric parameters (Å, º)

**Table:**

O1—C1	1.2077 (14)	C17—H17B	0.9600
O2—C1	1.3536 (14)	C17—H17C	0.9600
O2—C11	1.4030 (15)	C17—H17D	0.9600
N1—C1	1.3462 (15)	C17—H17E	0.9600
N1—C21	1.3982 (15)	C17—H17F	0.9600
N1—H1	0.875 (16)	C21—C26	1.3832 (16)
C11—C12	1.378 (2)	C21—C22	1.3894 (16)
C11—C16	1.3702 (19)	C22—N23	1.3270 (17)
C12—C13	1.394 (2)	C22—H22	0.9300
C12—C17	1.499 (2)	N23—C24	1.3317 (19)
C13—C14	1.370 (2)	C24—C25	1.373 (2)
C13—H13	0.9300	C24—H24	0.9300
C14—C15	1.368 (3)	C25—C26	1.3757 (18)
C14—H14	0.9300	C25—H25	0.9300
C15—C16	1.371 (2)	C26—H26	0.9300
C15—H15	0.9300	O1W—H1W	0.85 (2)
C16—H16	0.9300	O1W—H2W	0.86 (2)
C17—H17A	0.9600		
			
C1—O2—C11	116.62 (9)	H17A—C17—H17D	141.1
C1—N1—C21	125.36 (10)	H17B—C17—H17D	56.3
C1—N1—H1	115.3 (10)	H17C—C17—H17D	56.3
C21—N1—H1	118.9 (10)	C12—C17—H17E	109.5
O1—C1—N1	127.48 (11)	H17A—C17—H17E	56.3
O1—C1—O2	123.62 (11)	H17B—C17—H17E	141.1
N1—C1—O2	108.90 (10)	H17C—C17—H17E	56.3
C16—C11—C12	122.90 (13)	H17D—C17—H17E	109.5
C16—C11—O2	118.51 (12)	C12—C17—H17F	109.5
C12—C11—O2	118.45 (12)	H17A—C17—H17F	56.3
C11—C12—C13	116.07 (14)	H17B—C17—H17F	56.3
C11—C12—C17	121.12 (15)	H17C—C17—H17F	141.1
C13—C12—C17	122.80 (16)	H17D—C17—H17F	109.5
C14—C13—C12	121.78 (15)	H17E—C17—H17F	109.5
C14—C13—H13	119.1	C26—C21—C22	117.50 (11)
C12—C13—H13	119.1	C26—C21—N1	124.93 (10)
C15—C14—C13	120.14 (15)	C22—C21—N1	117.52 (10)
C15—C14—H14	119.9	N23—C22—C21	123.96 (12)
C13—C14—H14	119.9	N23—C22—H22	118.0
C14—C15—C16	119.80 (15)	C21—C22—H22	118.0
C14—C15—H15	120.1	C22—N23—C24	117.53 (11)
C16—C15—H15	120.1	N23—C24—C25	122.67 (12)
C11—C16—C15	119.32 (14)	N23—C24—H24	118.7
C11—C16—H16	120.3	C25—C24—H24	118.7
C15—C16—H16	120.3	C24—C25—C26	119.61 (12)
C12—C17—H17A	109.5	C24—C25—H25	120.2
C12—C17—H17B	109.5	C26—C25—H25	120.2
H17A—C17—H17B	109.5	C25—C26—C21	118.72 (11)
C12—C17—H17C	109.5	C25—C26—H26	120.6
H17A—C17—H17C	109.5	C21—C26—H26	120.6
H17B—C17—H17C	109.5	H1W—O1W—H2W	104.2 (18)
C12—C17—H17D	109.5		
			
C21—N1—C1—O1	−4.1 (2)	C12—C11—C16—C15	0.1 (2)
C21—N1—C1—O2	175.66 (11)	O2—C11—C16—C15	175.69 (12)
C11—O2—C1—O1	−8.96 (18)	C14—C15—C16—C11	−0.1 (2)
C11—O2—C1—N1	171.27 (11)	C1—N1—C21—C26	−24.33 (19)
C1—O2—C11—C16	94.57 (14)	C1—N1—C21—C22	158.06 (12)
C1—O2—C11—C12	−89.69 (14)	C26—C21—C22—N23	0.29 (19)
C16—C11—C12—C13	0.09 (19)	N1—C21—C22—N23	178.08 (12)
O2—C11—C12—C13	−175.46 (11)	C21—C22—N23—C24	0.2 (2)
C16—C11—C12—C17	−179.23 (15)	C22—N23—C24—C25	−0.2 (2)
O2—C11—C12—C17	5.22 (19)	N23—C24—C25—C26	−0.2 (2)
C11—C12—C13—C14	−0.3 (2)	C24—C25—C26—C21	0.63 (19)
C17—C12—C13—C14	178.99 (16)	C22—C21—C26—C25	−0.67 (17)
C12—C13—C14—C15	0.3 (2)	N1—C21—C26—C25	−178.28 (11)
C13—C14—C15—C16	−0.1 (2)		

### (CmoM) 2-Methylphenyl N-(pyridin-3-yl)carbamate monohydrate Hydrogen-bond geometry (Å, º)

**Table:**

*D*—H···*A*	*D*—H	H···*A*	*D*···*A*	*D*—H···*A*
N1—H1···O1*W*	0.875 (16)	1.953 (16)	2.8274 (14)	179.1 (15)
O1*W*—H1*W*···O1^i^	0.85 (2)	2.06 (2)	2.9126 (15)	173.2 (18)
O1*W*—H2*W*···N23^ii^	0.86 (2)	1.97 (3)	2.8266 (16)	170.6 (19)
C26—H26···O1	0.93	2.43	2.9337 (15)	114

Symmetry codes: (i) *x*, −*y*+3/2, *z*+1/2; (ii) −*x*+1, −*y*+2, −*z*.

### (CmoBr) 2-Bromophenyl N-(pyridin-3-yl)carbamate monohydrate Crystal data

**Table:**

C_12_H_9_BrN_2_O_2_·H_2_O	*D*_x_ = 1.627 Mg m^−^^3^
*M**_r_* = 311.14	Melting point: 359.5 K
Monoclinic, *P*2_1_/*c*	Mo *K*α radiation, λ = 0.71073 Å
*a* = 10.9036 (4) Å	Cell parameters from 3330 reflections
*b* = 13.0518 (3) Å	θ = 2.3–27.8°
*c* = 8.9804 (3) Å	µ = 3.24 mm^−^^1^
β = 96.460 (3)°	*T* = 294 K
*V* = 1269.90 (7) Å^3^	Plate, colourless
*Z* = 4	0.35 × 0.20 × 0.04 mm
*F*(000) = 624	

### (CmoBr) 2-Bromophenyl N-(pyridin-3-yl)carbamate monohydrate Data collection

**Table:**

Agilent Xcalibur Sapphire3 Gemini Ultra diffractometer	*R*_int_ = 0.029
Radiation source: Enhance (Mo) X-ray Source	θ_max_ = 27.9°, θ_min_ = 2.4°
ω scans	*h* = −13→11
Absorption correction: analytical (ABSFAC; Clark and Reid, 1998)	*k* = −16→16
*T*_min_ = 0.398, *T*_max_ = 0.844	*l* = −8→11
9904 measured reflections	3330 standard reflections every 60 min
2811 independent reflections	intensity decay: 1%
1881 reflections with *I* > 2σ(*I*)	

### (CmoBr) 2-Bromophenyl N-(pyridin-3-yl)carbamate monohydrate Refinement

**Table:**

Refinement on *F*^2^	Primary atom site location: structure-invariant direct methods
Least-squares matrix: full	Secondary atom site location: inferred from neighbouring sites
*R*[*F*^2^ > 2σ(*F*^2^)] = 0.046	Hydrogen site location: mixed
*wR*(*F*^2^) = 0.106	H atoms treated by a mixture of independent and constrained refinement
*S* = 1.03	*w* = 1/[σ^2^(*F*_o_^2^) + (0.0324*P*)^2^ + 1.3864*P*] where *P* = (*F*_o_^2^ + 2*F*_c_^2^)/3
2811 reflections	(Δ/σ)_max_ < 0.001
175 parameters	Δρ_max_ = 0.68 e Å^−^^3^
0 restraints	Δρ_min_ = −0.64 e Å^−^^3^

### (CmoBr) 2-Bromophenyl N-(pyridin-3-yl)carbamate monohydrate Special details

**Table:**

Geometry. All e.s.d.'s (except the e.s.d. in the dihedral angle between two l.s. planes) are estimated using the full covariance matrix. The cell e.s.d.'s are taken into account individually in the estimation of e.s.d.'s in distances, angles and torsion angles; correlations between e.s.d.'s in cell parameters are only used when they are defined by crystal symmetry. An approximate (isotropic) treatment of cell e.s.d.'s is used for estimating e.s.d.'s involving l.s. planes.

### (CmoBr) 2-Bromophenyl N-(pyridin-3-yl)carbamate monohydrate Fractional atomic coordinates and isotropic or equivalent isotropic displacement parameters (Å^2^)

**Table:**

	*x*	*y*	*z*	*U*_iso_*/*U*_eq_	
Br1	0.04274 (5)	0.72890 (3)	−0.00738 (5)	0.0794 (2)	
O1	0.3437 (2)	0.58402 (17)	0.0455 (3)	0.0543 (6)	
O2	0.2663 (2)	0.68665 (17)	0.2147 (3)	0.0514 (6)	
N1	0.4156 (3)	0.7464 (2)	0.0937 (3)	0.0447 (7)	
H1	0.396 (3)	0.794 (2)	0.127 (3)	0.025 (9)*	
C1	0.3436 (3)	0.6650 (2)	0.1092 (4)	0.0423 (7)	
C11	0.1718 (3)	0.6166 (2)	0.2272 (4)	0.0444 (8)	
C12	0.0609 (3)	0.6262 (2)	0.1409 (4)	0.0490 (9)	
C13	−0.0350 (4)	0.5605 (3)	0.1619 (5)	0.0609 (10)	
H13	−0.1107	0.5671	0.1037	0.073*	
C14	−0.0178 (4)	0.4857 (3)	0.2692 (5)	0.0644 (11)	
H14	−0.0820	0.4411	0.2835	0.077*	
C15	0.0934 (4)	0.4761 (3)	0.3554 (5)	0.0668 (11)	
H15	0.1036	0.4247	0.4273	0.080*	
C16	0.1896 (4)	0.5401 (3)	0.3382 (4)	0.0559 (9)	
H16	0.2646	0.5335	0.3979	0.067*	
C21	0.5132 (3)	0.7507 (2)	0.0050 (3)	0.0398 (7)	
C22	0.5498 (3)	0.8458 (3)	−0.0405 (4)	0.0499 (9)	
H22	0.5062	0.9029	−0.0134	0.060*	
N23	0.6435 (3)	0.8609 (2)	−0.1207 (3)	0.0563 (8)	
C24	0.7041 (4)	0.7790 (3)	−0.1586 (4)	0.0584 (10)	
H24	0.7698	0.7880	−0.2150	0.070*	
C25	0.6750 (3)	0.6817 (3)	−0.1188 (4)	0.0525 (9)	
H25	0.7201	0.6262	−0.1479	0.063*	
C26	0.5787 (3)	0.6667 (2)	−0.0356 (4)	0.0461 (8)	
H26	0.5578	0.6011	−0.0070	0.055*	
O1W	0.3476 (3)	0.9335 (2)	0.2214 (4)	0.0694 (9)	
H1W	0.345 (4)	0.931 (3)	0.304 (5)	0.071 (16)*	
H2W	0.347 (3)	0.986 (3)	0.199 (4)	0.048 (12)*	

### (CmoBr) 2-Bromophenyl N-(pyridin-3-yl)carbamate monohydrate Atomic displacement parameters (Å^2^)

**Table:**

	*U*^11^	*U*^22^	*U*^33^	*U*^12^	*U*^13^	*U*^23^
Br1	0.1013 (4)	0.0583 (3)	0.0771 (3)	0.0123 (2)	0.0035 (3)	0.0144 (2)
O1	0.0693 (17)	0.0377 (13)	0.0605 (15)	−0.0094 (11)	0.0279 (13)	−0.0112 (11)
O2	0.0597 (15)	0.0439 (13)	0.0542 (15)	−0.0133 (11)	0.0228 (12)	−0.0137 (11)
N1	0.0549 (19)	0.0291 (15)	0.0519 (17)	−0.0018 (13)	0.0142 (14)	−0.0053 (12)
C1	0.047 (2)	0.0390 (17)	0.0419 (18)	−0.0009 (15)	0.0090 (15)	0.0001 (14)
C11	0.053 (2)	0.0415 (17)	0.0416 (19)	−0.0033 (15)	0.0190 (17)	−0.0089 (14)
C12	0.062 (2)	0.0411 (18)	0.046 (2)	0.0064 (16)	0.0164 (18)	−0.0038 (14)
C13	0.053 (2)	0.064 (2)	0.067 (3)	−0.0051 (19)	0.015 (2)	−0.013 (2)
C14	0.066 (3)	0.063 (2)	0.070 (3)	−0.017 (2)	0.030 (2)	−0.007 (2)
C15	0.087 (3)	0.061 (2)	0.057 (2)	−0.006 (2)	0.026 (2)	0.0085 (19)
C16	0.055 (2)	0.062 (2)	0.053 (2)	−0.0061 (18)	0.0146 (18)	−0.0086 (18)
C21	0.0423 (18)	0.0383 (18)	0.0381 (16)	−0.0044 (13)	0.0013 (14)	0.0021 (13)
C22	0.052 (2)	0.0381 (18)	0.060 (2)	−0.0024 (15)	0.0055 (18)	0.0043 (15)
N23	0.056 (2)	0.0505 (18)	0.063 (2)	−0.0095 (15)	0.0111 (16)	0.0128 (14)
C24	0.049 (2)	0.070 (3)	0.058 (2)	−0.0043 (19)	0.0125 (18)	0.0058 (19)
C25	0.045 (2)	0.056 (2)	0.057 (2)	0.0053 (17)	0.0091 (17)	−0.0036 (17)
C26	0.050 (2)	0.0353 (17)	0.053 (2)	−0.0005 (15)	0.0047 (17)	0.0022 (14)
O1W	0.116 (3)	0.0386 (16)	0.056 (2)	0.0123 (16)	0.0216 (19)	0.0024 (14)

### (CmoBr) 2-Bromophenyl N-(pyridin-3-yl)carbamate monohydrate Geometric parameters (Å, º)

**Table:**

Br1—C12	1.884 (3)	C15—H15	0.9300
O1—C1	1.201 (4)	C16—H16	0.9300
O2—C1	1.367 (4)	C21—C22	1.380 (4)
O2—C11	1.391 (4)	C21—C26	1.380 (4)
N1—C1	1.337 (4)	C22—N23	1.329 (4)
N1—C21	1.401 (4)	C22—H22	0.9300
N1—H1	0.74 (3)	N23—C24	1.322 (5)
C11—C12	1.367 (5)	C24—C25	1.366 (5)
C11—C16	1.409 (5)	C24—H24	0.9300
C12—C13	1.381 (5)	C25—C26	1.369 (5)
C13—C14	1.370 (6)	C25—H25	0.9300
C13—H13	0.9300	C26—H26	0.9300
C14—C15	1.369 (6)	O1W—H1W	0.75 (4)
C14—H14	0.9300	O1W—H2W	0.71 (4)
C15—C16	1.363 (5)		
			
C1—O2—C11	116.1 (2)	C14—C15—H15	119.2
C1—N1—C21	125.7 (3)	C15—C16—C11	117.9 (4)
C1—N1—H1	116 (2)	C15—C16—H16	121.1
C21—N1—H1	118 (2)	C11—C16—H16	121.1
O1—C1—N1	128.1 (3)	C22—C21—C26	117.5 (3)
O1—C1—O2	123.1 (3)	C22—C21—N1	117.9 (3)
N1—C1—O2	108.8 (3)	C26—C21—N1	124.6 (3)
C12—C11—O2	120.7 (3)	N23—C22—C21	124.0 (3)
C12—C11—C16	120.6 (3)	N23—C22—H22	118.0
O2—C11—C16	118.6 (3)	C21—C22—H22	118.0
C11—C12—C13	120.1 (3)	C24—N23—C22	117.2 (3)
C11—C12—Br1	118.8 (3)	N23—C24—C25	123.1 (3)
C13—C12—Br1	121.1 (3)	N23—C24—H24	118.4
C14—C13—C12	119.5 (4)	C25—C24—H24	118.4
C14—C13—H13	120.2	C24—C25—C26	119.4 (3)
C12—C13—H13	120.2	C24—C25—H25	120.3
C15—C14—C13	120.4 (4)	C26—C25—H25	120.3
C15—C14—H14	119.8	C25—C26—C21	118.8 (3)
C13—C14—H14	119.8	C25—C26—H26	120.6
C16—C15—C14	121.6 (4)	C21—C26—H26	120.6
C16—C15—H15	119.2	H1W—O1W—H2W	109 (5)
			
C21—N1—C1—O1	−4.6 (6)	C14—C15—C16—C11	−0.7 (5)
C21—N1—C1—O2	174.6 (3)	C12—C11—C16—C15	0.5 (5)
C11—O2—C1—O1	−10.8 (5)	O2—C11—C16—C15	176.0 (3)
C11—O2—C1—N1	169.9 (3)	C1—N1—C21—C22	157.9 (3)
C1—O2—C11—C12	−88.3 (4)	C1—N1—C21—C26	−24.6 (5)
C1—O2—C11—C16	96.3 (3)	C26—C21—C22—N23	0.1 (5)
O2—C11—C12—C13	−175.4 (3)	N1—C21—C22—N23	177.8 (3)
C16—C11—C12—C13	−0.1 (5)	C21—C22—N23—C24	0.2 (6)
O2—C11—C12—Br1	5.3 (4)	C22—N23—C24—C25	−0.2 (6)
C16—C11—C12—Br1	−179.3 (2)	N23—C24—C25—C26	−0.1 (6)
C11—C12—C13—C14	−0.3 (5)	C24—C25—C26—C21	0.4 (5)
Br1—C12—C13—C14	178.9 (3)	C22—C21—C26—C25	−0.4 (5)
C12—C13—C14—C15	0.2 (5)	N1—C21—C26—C25	−177.8 (3)
C13—C14—C15—C16	0.3 (6)		

### (CmoBr) 2-Bromophenyl N-(pyridin-3-yl)carbamate monohydrate Hydrogen-bond geometry (Å, º)

**Table:**

*D*—H···*A*	*D*—H	H···*A*	*D*···*A*	*D*—H···*A*
N1—H1···O1*W*	0.74 (3)	2.10 (3)	2.832 (4)	177 (3)
O1*W*—H1*W*···O1^i^	0.75 (4)	2.18 (5)	2.924 (4)	177 (5)
O1*W*—H2*W*···N23^ii^	0.71 (4)	2.13 (4)	2.837 (4)	175 (4)
C26—H26···O1	0.93	2.44	2.946 (4)	114

Symmetry codes: (i) *x*, −*y*+3/2, *z*+1/2; (ii) −*x*+1, −*y*+2, −*z*.
